
JOURNAL INFORMATION
==============================
NLM Title Abbreviation: Evol Appl
Journal ID: Evol Appl
NLM Journal ID: 101461828
Journal ID: eva

Evolutionary Applications

EISSN: 1752-4571
Publisher: Wiley

ARTICLE INFORMATION
==============================
PMCID: PMC4055172
DOI: 10.1111/eva.12104
Article version: 1
Subjects: Issue Information

Issue Information

Print publication date: 2014 May
Electronic publication date: 2014 May 27
Volume: 7
Issue: 5
First page: i
Last page: i
Copyright: © 2014 John Wiley & Sons Ltd
Copyright year: 2014
License: This is an open access article under the terms of the Creative Commons Attribution License, which permits use, distribution and reproduction in any medium, provided the original work is properly cited.
License URL: https://creativecommons.org/licenses/by/3.0/

==============================
### Evolutionary Applications

Open Access

*Editor-in-Chief*
**LOUIS BERNATCHEZ**
*Université Laval, Quebec. Canada*

*Founding Editor*
**MICHELLE TSENG**
*University of British Columbia, Canada*

*Associate Editors*
**SCOTT CARROLL**
*Institute for Contemporary Evolution/UC Davis, USA*
**BERNARD CRESPI**
*Simon Fraser University, Canada*
**ARNAUD ESTOUP**
*CBGP, Montpellier, France*
**DYLAN FRASER**
*Concordia University, Canada*
**ZACH GOMPERT**
*Utah State University, USA*
**ANDREW HENDRY**
*McGill University, Canada*
**REES KASSEN**
*University of Ottawa, Canada*
**BRITT KOSKELLA**
*University of Exeter, UK*
**INGRID PARKER**
*University of California, Santa Cruz, USA*
**CRAIG PRIMMER**
*University of Turku, Finland*
**ANDREW READ**
*Penn State University, USA*
**OUTI SAVOLAINEN**
*University of Oulu, Finland*
**CARLA SGRO**
*Monash University, Australia*
**PETE THRALL**
*CSIRO, Australia*
**FRÉDÉRIC THOMAS**
*CNRS/IRD, Montpellier, France*

*Editorial Office Support*
**WEI-MUN CHAN**
*Wiley Blackwell, Oxford, UK*

*Advisory Board*
**S. Aitken** *University of British Columbia, Canada*
**F. Allendorf** *University of Montana, USA*
**S. Barrett** *University of Toronto, Canada*
**T. Day** *Queens University, Canada*
**N. Ellstrand** *University of California, Riverside, USA*
**G. Gilchrist** *College of William and Mary / NSF, USA*
**J. Hellmann** *University of Notre Dame, USA*
**A. Hoffman** *University of Melbourne, Australia*
**J. Hutchings** *Dalhousie University, Canada*
**P. Jarne** *CEFE, Montpellier, France*
**N. Kane** *University of British Columbia, Canada*
**B. Koskella** *University of Exeter, UK*
**J. A. Merilä** *University of Helsinki, Finland*
**Y. Michalakis** *IRD, Montpellier, France*
**J. Myers** *University of British Columbia, Canada*
**I. Olivieri** *ISEM, Montpellier, France*
**L. Rieseberg** *University of British Columbia, Canada / Indiana University, USA*
**T. Smith** *University of California, Los Angeles, USA*
**S. Strauss** *University of California, Davis, USA*
**J. Webster** *Imperial College London, UK*

### Aims and Scope

*Evolutionary Applications* is a fully peer reviewed open access journal. It publishes papers that utilize concepts from evolutionary biology to address biological questions of health, social and economic relevance. Papers are expected to employ evolutionary concepts or methods to make contributions to areas such as (but not limited to): medicine, agriculture, forestry, exploitation and management (fisheries and wildlife), aquaculture, conservation biology, environmental sciences (including climate change and invasion biology), microbiology, and toxicology. All taxonomic groups are covered from microbes, fungi, plants and animals. In order to better serve the community, we also now strongly encourage submissions of papers making use of modern molecular and genetic methods (population and functional genomics, transcriptomics, proteomics, epigenetics, quantitative genetics, association and linkage mapping) to address important questions in any of these disciplines and in an applied evolutionary framework. Theoretical, empirical, synthesis or perspective papers are welcome.

*Evolutionary Applications* is a Wiley Open Access journal, one of a new series of peer-reviewed titles publishing quality research with speed and efficiency. For further information visit the Wiley Open Access website at http://www.wileyopenaccess.com

### Open Access and Copyright

All articles published by *Evolutionary Applications* are fully open access: immediately freely available to read, download and share. All articles accepted from 14 August 2012 are published under the terms of the Creative Commons Attribution License. All articles accepted before this date were published under a Creative Commons Attribution Non-Commercial License. The Creative Commons Attribution License permits use, distribution and reproduction in any medium, provided the original work is properly cited and allows the commercial use of published articles. Copyright on any research article in a journal published by *Evolutionary Applications* is retained by the author(s). Authors grant Wiley a license to publish the article and identify itself as the original publisher. Authors also grant any third party the right to use the article freely as long as its integrity is maintained and its original authors, citation details and publisher are identified. Further information about open access license and copyright can be found at http://www.wileyopenaccess.com/details/content/12f25db4c87/Copyright–License.html.

Special requests should be addressed to permissionsuk@wiley.com. Print reprints of Wiley Open Access articles can be purchased from corporatesales@wiley.com.

### Disclaimer

The Publisher and Editors cannot be held responsible for errors or any consequences arising from the use of information contained in this journal; the views and opinions expressed do not necessarily reflect those of the Publisher and Editors, neither does the publication of advertisements constitute any endorsements by the Publisher and Editors of the products advertised.

Wiley Open Access articles posted to repositories or websites are without warranty from Wiley of any kind, either express or implied, including, but not limited to, warranties of merchantability, fitness for a particular purpose, or non-infringement. To the fullest extent permitted by law Wiley disclaims all liability for any loss or damage arising out of, or in connection, with the use of or inability to use the content.

*Front cover:* Courtship display of male Houbara bustard. Photo Credit: Y. Hingrat (RENECO).
